# Sleep quality and neurohormonal and psychophysiological accompanying factors in adolescents with depressive disorders: study protocol

**DOI:** 10.1192/bjo.2022.29

**Published:** 2022-03-03

**Authors:** Rebekka Krempel, Daniel Schleicher, Irina Jarvers, Angelika Ecker, Romuald Brunner, Stephanie Kandsperger

**Affiliations:** Clinic of Child and Adolescent Psychiatry, Psychosomatics and Psychotherapy, University of Regensburg, Germany; Clinic of Child and Adolescent Psychiatry, Psychosomatics and Psychotherapy, University of Regensburg, Germany; Clinic of Child and Adolescent Psychiatry, Psychosomatics and Psychotherapy, University of Regensburg, Germany; Clinic of Child and Adolescent Psychiatry, Psychosomatics and Psychotherapy, University of Regensburg, Germany; Clinic of Child and Adolescent Psychiatry, Psychosomatics and Psychotherapy, University of Regensburg, Germany; Clinic of Child and Adolescent Psychiatry, Psychosomatics and Psychotherapy, University of Regensburg, Germany

**Keywords:** Adolescence, depression, sleep accelerometry, cortisol awakening response, alpha-amylase

## Abstract

**Background:**

Depressive disorders are common mental health problems during adolescence. Many adolescents with depression describe difficulties with sleeping. Findings of previous studies regarding changes in objective sleep quality in adolescents with depressive disorders are heterogeneous.

**Aims:**

This study aims to investigate differences in objective and subjective sleep quality between adolescents with depressive disorders and healthy peers, and to evaluate if potential changes in sleep occur concurrently with changes in the release of cortisol and alpha-amylase after awakening.

**Method:**

This non-interventional parallel study examines correlations between depressive disorders, sleep quality and release of stress hormones. Sleep quality in the past 2 weeks, severity of depressive symptoms, psychiatric comorbidities and stress response of 30 adolescents with depressive disorders and 30 healthy controls (*N* = 60) are assessed via questionnaires. In participants’ home environments, the objective sleep quality of seven consecutive nights is measured by sleep accelerometry. After awakening, participants answer sleep questionnaires to examine the subjective sleep quality of those nights. Furthermore, salivary cortisol and alpha-amylase are measured three times after awakening (+0 min, +30 min and +45 min after awakening).

**Conclusions:**

Sleep is an important factor for prognosis and well-being in adolescents with depression. The results of this study can be highly valuable to integrate a more detailed examination of sleep quality and sleeping impairments in the treatment of adolescent depressive disorders.

## Depression as a global health problem

According to the World Health Organization, depression is the ‘leading course of disability worldwide’ and ‘a major contributor to the overall global burden of disease’, with more than 264 million people of all ages with depressive disorders.^1^ Affecting performance at school or work, as well as relationships and family constellations, depression threatens not only the global living environment of individuals, but of society as a whole.^2^

## Depressive disorders in adolescence

Depressive disorders are common mental health problems during adolescence. In a survey conducted in the USA, a lifetime prevalence of about 12% was found for the adolescent sample, and prevalence nearly doubled from early (13/14 years: 8.4%) to late adolescence (17/18 years: 15.4%).^3^ Additionally, the number of adolescents with depressive symptoms is even higher when considering the high number of adolescents with subthreshold depression, with a lifetime prevalence of up to 20% at the age of 18 years.^4^

Depressive disorders during adolescence are associated with more burdensome circumstances in adulthood, including mental health problems, suicidal ideation or attempts, reduced educational attainment and economic status, lower relationship quality and higher levels of conflicts and violence.^5^ This underlines the importance of a deeper understanding of the aetiology and severity of depressive disorder.

## Sleep disturbance in patients with depressive disorder

Sleep disturbance constitutes an important symptom of depressive disorders, and it gains even greater importance when considering that it may increase the risk of suicidal behaviour among adolescents.^6^ Delayed sleep onset and decreased sleep efficiency, combined with changes in rapid eye movement (e.g. shorter latency and increased activity, density and amount of rapid eye movement sleep) and slow-wave sleep (e.g. decreased sleep amount), are frequent findings from studies assessing sleep in adults with depression.^7^ Regarding paediatric depression, findings are far more heterogeneous. In his review dealing with sleep disturbance in paediatric depression, Rao considered 24 electroencephalogram studies conducted in children and adolescents.^7^ A prolonged sleep latency was found in eight of these studies, and 15 studies found no differences. A decrease in sleep efficiency was found in three of the studies, and 19 studies found no differences.^7^ As sleep underlies multifarious influences in the context of development, the heterogenous study situation could reflect the complexity of sleep quality and its components during the developmental period.^7^ Examination of sleep latency and efficiency via accelerometry, as conducted in this study, has been shown to be a promising approach in previous research.^8,9^

In addition to objective measures of sleep quality, the burden of impaired subjective sleep quality and sleep complaints caused by depression should be considered. Many studies in adults with depressive disorders have assessed subjective sleep quality, and findings show that over 80% of patients report sleep difficulties.^10^ Previous research showed that similar impairments can be found in adolescents with depressive disorders. Considering only subjective sleep assessments without objective sleep data, Gupta et al found impaired sleep quality, delayed sleep onset, shorter sleep duration, and a correlation of sleep quality with severity of depressive symptoms.^11^ These abovementioned impairments in subjective as well as objective sleep quality may lead to serious mental burden and an increased stress level in everyday life for many adolescents with depression; therefore, both measurements are important to consider.

## Cortisol

A hormone that plays a key role in the experience and management of stress is the glucocorticoid hormone cortisol. Its secretion from the adrenal cortex^12^ depends on stimulation by adrenocorticotropin released by the anterior pituitary gland (hypothalamus–pituitary–adrenal (HPA) axis activation), and follows a characteristic diurnal rhythm with a peak in the early morning.^12^ Activation of the HPA axis and the ability to adjust cortisol release is an important contributor for adapting to stressful life events, and providing energy and physical resources to cope with the demanding situation.^13^ Chronic activation, however, is linked to various somatic and mental health problems.^13,14^ In our study, we focus on the strength of the cortisol peak within 45 min after awakening, known as the cortisol awakening response (CAR).^13^ Characteristics of the CAR include a precipitous increase after awakening, a maximum within about 30 min after awakening and a subsequent decline.^12^ CAR constitutes a ‘reliable biological marker for adrenocortical activity’.^15^ An increased as well as a decreased CAR is associated with stress and multifarious health problems, such as autoimmune, allergic and psychiatric illnesses.^13,16^ Several previous studies examined changes in the CAR in differently structured samples of adults with depressive disorders. Bhagwagar et al^17^ observed an increased CAR in adult patients with depressive disorders; Vreeburg et al^18^ found similar results in patients with remitted and current depressive symptoms. Additionally, Stroud et al^16^ examined an interaction between larger CAR and a greater extent of depressive symptoms, whereas Huber et al^19^ found an attenuated CAR in psychotherapy in-patients with depression. In adults with subclinical depressive symptoms, an attenuation of the CAR was found by Mangold et al.^20^ According to Elder et al,^21^ the use of antidepressant medication may be a contributing factor to the inconsistency of findings, and so we include only participants who are not taking antidepressant medications. Regarding our study cohort, previous research with adolescent study samples is of particular interest. In young girls with depressive disorders, Schmidt et al^14^ found a significantly higher CAR. However, this study did not exclude adolescents on antidepressant medications; therefore, our study design with a medication-free sample adds important value to the current study situation.

## Alpha-amylase

Another measurement for the activation of physiological stress systems is the enzyme alpha-amylase. It is secreted by the sympathetic and parasympathetic innervated salivary glands, and constitutes an important biomarker of autonomic nervous system activity with a sensitive responsiveness to acute stress situations.^22^ Examining alpha-amylase levels of adult patients with depression, recent studies found elevated salivary alpha-amylase levels after awakening, and provided evidence that alpha-amylase may be a promising biomarker for depressive disorders.^23^ Regarding paediatric depression, very few studies have examined alpha-amylase levels in children and adolescents with depression.^24^ To our knowledge, no study has focused on the reactivity of alpha-amylase with various measurements after awakening in adolescent patients.

The examination of alpha-amylase provides further information regarding the stress system dysregulation compared with an exclusive assessment of cortisol,^25^ which makes a parallel measurement of these two biomarker a useful approach.

## Interrelation between sleep, depressive symptoms and psychophysiological correlates

When examining the link between sleep quality, depressive symptoms and psychophysiological correlates, the interrelations are multifactorial and the direction is partially unclear. Fang et al found that the association between sleep and depressive symptoms in adult patients with depression is bidirectional, with sleep impairment being a comorbidity as well as a prodromal sign of a depressive disorder.^26^ In adolescent patients, previous studies reported a connection between sleep problems and later depressive symptoms,^27^ but prior research also identified that higher severity of depressive symptoms was associated with poorer sleep quality.^28^

There are also indications that sleep and stress biomarkers are linked bidirectionally. A hyperactivity of the HPA axis can influence sleep architecture and decrease sleep quality, but sleep impairments also negatively affect functioning of the HPA axis.^29^ A previous study observed a positive association between sleep duration and levels of cortisol, but no association between subjective sleep quality and cortisol levels at awakening, whereas levels of alpha-amylase at awakening were not significantly associated with sleep duration, but a worse subjective sleep quality was associated with higher alpha-amylase levels at awakening.^30^

Much research was performed examining the relationship between cortisol and depressive symptoms in adolescents and adults. In this context, the release of cortisol is considered a heritable trait,^31^ with a connection to current^14^ and future^16^ depressive symptoms. Referring to alpha-amylase, prior research found elevated alpha-amylase levels at awakening in adult patients with depression,^23^ but first investigations in an adolescent sample did not confirm this alpha-amylase morning elevation in adolescent patients with depression.^24^

## Implications for the planned study

According to Nater et al, the simultaneous examination of HPA axis indicators (cortisol) and biomarkers of the autonomic nervous system (alpha-amylase) is a recommended approach, providing further information about psychobiological stress responses.^22^ By repeatedly analysing cortisol as well as alpha-amylase levels after awakening in adolescents with depression and healthy controls, we are able to assess neuroendocrinological biomarkers that may play a key role in many depressive symptoms, including sleep impairments. Based on the secretion mechanism and physiological compartment, we do not expect a correlation between cortisol and alpha-amylase secretion.

Additionally, our study design enables us to examine sleep and psychophysiological correlates as close to reality as possible. Examining sleep in participants’ home environments^10^ and the simple and non-invasive wearing of a watch-like accelerometer barely influences participants’ natural sleeping habits,^9^ and makes a realistic assessment of subjective as well as objective sleep quality possible. Together with saliva sampling in a familiar environment,^32^ these ambulatory assessment methods lead to a high ecological validity of the study findings.

## Study aim

Investigating the relationship between sleep quality and depressive symptoms, our study's goal is to compare objective measurements and subjective assessments of sleep quality between adolescents with depressive disorders and healthy peers. Another focus of our study lies in the examination of salivary cortisol and alpha-amylase releasing patterns after awakening as a potentially correlated factor in altered sleeping patterns.

## Hypotheses

We hypothesise that adolescents with depressive disorders will show a lower objective sleep quality with lower sleep efficiency, longer onset latency, more frequent awakenings and longer wakefulness after sleep onset (WASO) than healthy controls. Based on previous studies, we expect the subjective sleep quality of participants with depression to be considerably lower than that of the control group (less satisfaction with sleep, worse mood after awakening, lower feeling of recreation after awakening, impression of frequent awakenings during the night and impaired subjective quality of dreams). Additionally, in accordance with Schmidt et al^14^ and Bauduin et al,^23^ we hypothesise an increased CAR and elevated alpha-amylase levels after awakening in the index group compared with the healthy control group.

## Method

### Study design

This is a non-interventional and parallelised study with a matched control group. The study was conducted monocentrally at the out-patient and day patient department of the Clinic of Child and Adolescent Psychiatry, Psychosomatics and Psychotherapy at the University of Regensburg, Germany. We choose the out-patient and day patient department to reduce sleep disturbance from the hospital environment or shared rooms expected for those patients admitted to hospital. By choosing a patient cohort that sleeps in their usual surroundings without external influences, we aim to focus on changes in sleep quality that can be attributed to the depressive disorder.

The study protocol conforms to the Standard Protocol Items: Recommendations for interventional Trials (SPIRIT) checklist.

### Ethics approval and consent to participate

The authors assert that all procedures contributing to this work comply with the ethical standards of the relevant national and institutional committees on human experimentation and with the Helsinki Declaration of 1975, as revised in 2008. All procedures involving human patients were approved by the ethics committee at the University of Regensburg. The committee consented to study execution on 19 February 2020, and the reference number is 20-1711-101.

Before any data was gathered, written informed consent was obtained from all participants and their legal guardians. Voluntary participation is mandatory and withdrawal from the study is possible at any time. Saliva samples given to the external laboratory for analysis are submitted, numbered and without any possibility of allocation.

This study is registered with the German Clinical Trials Register; the date of registration was 8 April 2020 and the registration number is DRKS00020907.

### Eligibility criteria

**Fig. 1:**
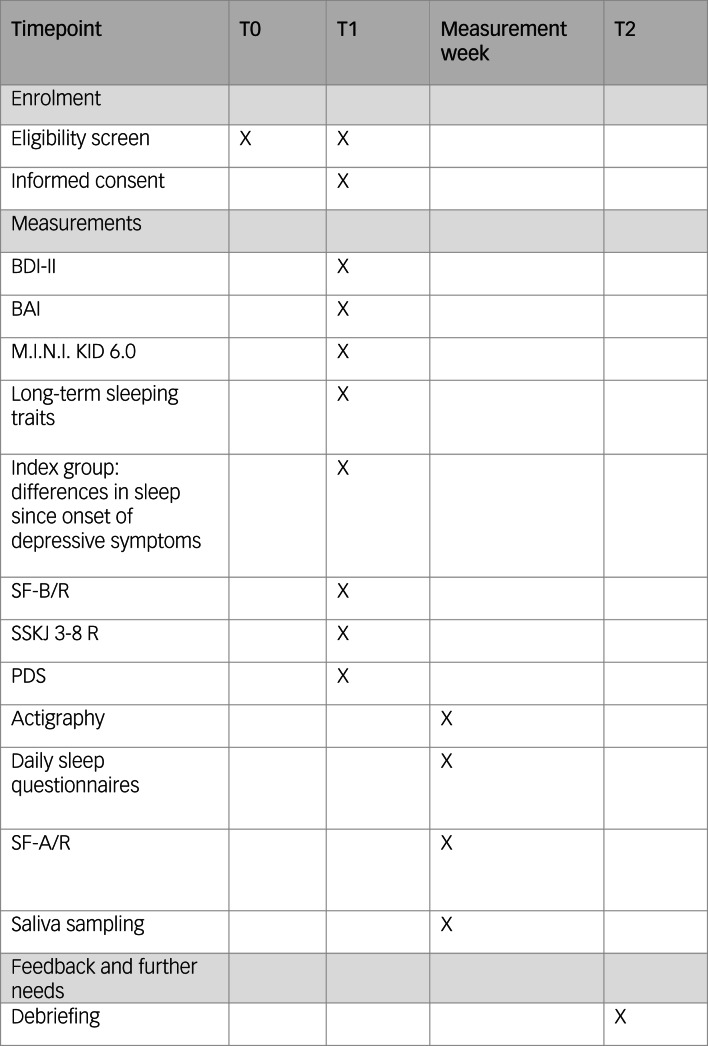
Study schedule. BAI, Beck Anxiety Inventory; BDI-II, Beck Depression Inventory-II; M.I.N.I KID 6.0, Mini-International Neuropsychiatric Interview for Children and Adolescents; PDS, Pubertal Development Scale; SF-A/R, Sleep Questionnaire A – revised version; SF-B/R, Sleep Questionnaire B – revised version; SSKJ 3-8 R, Questionnaire for the Measurement Stress and Coping in Children and Adolescents – revised version.

Eligibility criteria are screened twice before study participation: initially in the first contact via telephone, and a second time immediately before study participation at the first study appointment (eligibility screen, see Fig. 1).

#### Inclusion criteria

Children and adolescents between 12 and 18 years are eligible for participation. A sufficient understanding of the German language, as well as informed consent of both participants and legal guardians, is required to participate in the study.

For the index group, potential participants are those with depressive disorders according to the diagnostic criteria of the ICD-10.^33^ More precisely, we include the ICD-10 codes F32.0 (mild depressive episode), F32.1 (moderate depressive episode), F32.2 (severe depressive episode without psychotic symptoms), F33.0 (major depressive disorder, recurrent, mild), F33.1 (major depressive disorder, recurrent, moderate), F33.2 (major depressive disorder, recurrent, severe without psychotic features) and F43.21 (adjustment disorder with prolonged depressive reaction) in our index group.

For the control group, we include healthy adolescents matched by gender, age and pubertal status according to the Pubertal Development Scale (PDS). This shall align age- and gender-specific differences.^34^

#### Exclusion criteria

Potential participants who suffer from psychotic or acute suicidal conditions are excluded from study participation. Pregnancy, antidepressant or glucocorticoid medication, and neurological or endocrinological illnesses that may affect brain physiology or relevant endocrinological parameters are additional exclusion criteria. Moreover, those with cannabis consumption within the 3 months before study participation are excluded.

Furthermore, most psychiatric comorbidities constitute exclusion criteria. For the index group, we include anxiety disorders, as they appear cumulatively comorbid with depressive disorders.^35,36^ All other psychiatric comorbidities lead to exclusion.

For the control group, we exclude participants with any psychiatric disease, symptoms indicating a current psychiatric disease, a history of psychotherapeutic or psychiatric intervention, or a condition requiring therapeutic support. Potential participants excluded because of the latter criterion are offered therapeutic help if the indication is given.

### Recruitment

In the index group, participants with a diagnosed depressive disorder are consecutively recruited from the patient cohort of the out-patient department and day patient department of the Clinic of Child and Adolescent Psychiatry, Psychosomatics and Psychotherapy at the University of Regensburg, Germany.

To recruit healthy adolescents for the control group, we will announce the study and call for participation via mailing lists, websites and social media channels of our hospital.

In case of drop-out, recruitment will be continued using the abovementioned resources until the intended number of participants is achieved.

### Informed consent

No participant is included in the study unless written informed consent of the participants and legal guardians is given. If consent is revoked at any point during or after participation, all examinations of this study will be cancelled immediately for this participant.

### Measurements

#### Assessment of depressive symptoms, psychiatric comorbidities and stress responsiveness

Initially, all participants are screened and interviewed with the Mini-International Neuropsychiatric Interview for Children and Adolescents (M.I.N.I. KID 6.0)^37^ for psychiatric disorders (controls) or comorbidities (patients) (T1, see Fig. 1). The M.I.N.I. KID 6.0 interview is structured into 24 diagnostic modules assessing potential symptoms of psychiatric disorders according to the DSM-IV and ICD-10.

Depressive symptoms are assessed via the Beck Depression Inventory-II,^38^ which is one of the most commonly used self-rating scales to measure depressive symptoms^39^ from the age of 13 years (T1, see Fig. 1). Frequently observed symptoms are inquired for the past 2 weeks in 21 items, with four possible response options covering increasing symptom severity. As an adjustment for the younger study participants, we excluded item 21 (loss of interest in sexuality) in our assessment. Internal consistency for the German version is good (*α* ≥ 0.84), retest reliability is *r* ≥ 0.75 and discriminative validity is confirmed.^40^ The applicability to an adolescent sample was checked and validated.^41,42^

As symptoms of anxiety and anxiety disorders occur often concurrently with depressive disorders,^35,36^ we examine anxiety via the Beck Anxiety Inventory (BAI)^43^ (T1, see Fig. 1). The BAI consists of 21 items focusing on somatic symptoms of anxiety experienced within the past week.^44^ The German BAI showed reliable and valid psychometrics.^45^ For adolescent samples, the BAI showed at least acceptable psychometric properties in other language versions.^45,46^

Stable sleeping habits and needs will be assessed by a short self-designed self-report survey (T1, see Fig. 1). This survey assesses chronotype, the regularity of bedtimes, the duration of sleep needed to feel rested and the frequency of sleeping through school or work. Furthermore, it queries the usual bed and wake-up times for each school/working days and weekend days/holidays. The items used for this survey are based on the Sleep Habit Survey (SHS).^47^ For the subscales, the original SHS showed acceptable internal consistency, with *α* ranging from 0.70 to 0.79.^48^

Participants with a depressive disorder additionally answer a self-designed questionnaire examining potential differences in relevant aspects of sleep quality since the onset of depressive symptoms (T1, see Fig. 1). This questionnaire consists of eight items, in which the participants compare the need to sleep, sleep and dream quality, WASO, mood at awakening, time of awakening and personal relevance of the topic sleep before and after symptom onset, and evaluate changes on a five-point Likert scale.

Sleeping quality of the previous 2 weeks is evaluated with the Sleep Questionnaire B (SF-B/R)^49^ (T1, see Fig. 1). The SF-B/R is a validated 31-question survey. Five sleep indices can be calculated: difficulty falling asleep, difficulty staying asleep, premature awakening, general sleep characterisation and total sleep duration. In addition, seven factor scales (sleep quality, feeling of being refreshed after sleep, mental balance before sleep, feeling mentally exhausted before sleep, psychosomatic symptoms during the sleep phase, dream recall and sleep–wake regulation) can be assessed. The SF-B/R is designed for sleep research as well as clinical practice. Satisfactory reliability values >0.70 are shown with regard to the internal consistency of the factor levels and the retest reliability of the SF-B/R (2–4 weeks). With regard to convergent validity, medium correlations are found with construct-related domains, such as measures of well-being, depression and personality factors.^49^

Participants also complete the Stress and Coping Questionnaire for Children and Adolescents (SSKJ 3-8 R)^50^ (T1, see Fig. 1). The SSKJ 3-8 R is a self-report questionnaire based on the transactional model of stress and coping developed by Lazarus and Folkman, assessing several strategies to reduce negative emotions or to change conditions in problematic or negatively experienced situations.^51^ The first part comprises an assessment of vulnerability to different stressors on four-level visual analogue scales. The second part includes five- and three-point Likert scales evaluating the usage frequency of five different coping strategies.^51^ The German version of the SSKJ 3-8 R showed sufficient reliability and validity has been confirmed.^52,53^

Pubertal development is assessed with the use of the German adaption^54^ of the Pubertal Development Scale (PDS)^55^ as a self-report questionnaire (T1, see Fig. 1). Female participants asses the current status of breast development, pubic hair growth, growth in height and skin, and estimated level of development compared with peers. Male participants are asked to evaluate the start and level of beard growth, existence of puberty vocal changes, pubic hair growth, growth in height, and skin changes, and to compare their level of development with their peers.^54,55^ Measures of criterion validity as well as internal consistency have achieved acceptable values.^54^

#### Accelerometry

**Fig. 2:**
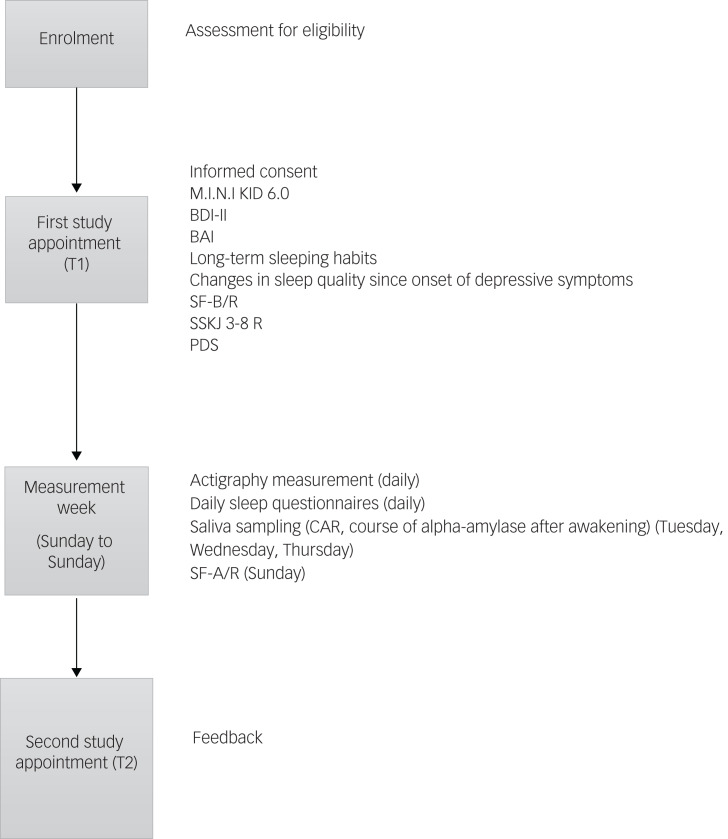
Study diagram. BAI, Beck Anxiety Inventory; BDI-II, Beck Depression Inventory-II; CAR, cortisol awakening response; M.I.N.I KID 6.0, Mini-International Neuropsychiatric Interview for Children and Adolescents; PDS, Pubertal Development Scale; SF-A/R, Sleep Questionnaire A – revised version; SF-B/R, Sleep Questionnaire B – revised version; SSKJ 3-8 R, Questionnaire for the Measurement Stress and Coping in Children and Adolescents – revised version.

For sleep measuring, we use the ActTrust 2 wrist actimeter (Condor Instruments) worn on the non-dominant wrist for seven consecutive nights, starting on Sunday evening (measurement week, see Fig. 2). Wearing and handling of this accelerometer is practiced by the adolescents and their legal guardians during the first study appointment. During the measurement week, participants put on the accelerometer when they go to bed in the evening. By pushing the event button, they record the time they intend to sleep and mark unexpected events or delays (for example, getting up again to go to the bathroom). While sleeping, data is recorded in 1-min intervals. The selected interval is the default of the manufacturer Condor Instruments, and is obligatory for the post-processing of sleep data. In the morning, wake-up time is recorded by pushing the event button again. Having marked the wake-up time, participants take the accelerometer off.

After participants return the accelerometer at the second study appointment, we check the recordings with the ActStudio software (Condor Instruments, São Paulo, Brazil, PC version 1.0.10, for Windows; see https://www.condorinst.com.br/en/actstudio-software/). In accordance with the standard procedure in sleep accelerometry, we manually identify the main sleep period.^56^ Bed time, get up time, time in bed, total sleep time, onset latency, sleep efficiency, WASO, awakenings and the intensity of blue light are provided by the software. Additionally, activity data during sleep is derived from the data, as increased motor activity is associated with impaired sleep quality.^9^

In the validation study, the ActTrust accelerometer achieved satisfactory results with a sensitivity of 95.69%, a specificity of 30.59%, an accuracy of 80.24%, a predictive value for sleep of 81.52% and a predictive value for wakefulness of 68.93%.^57^ Regarding the correlation analysis between accelerometry and polysomnography, *r* = 0.491 (*P* < 0.001) was observed for total sleep time, *r* = 0.499 (*P* < 0.001) for wake after sleep onset, *r* = 0.345 (*P* < 0.001) for sleep efficiency and no statistical significance was observed for sleep onset latency.^57^

#### Daily sleep questionnaires

To examine subjective sleep quality, participants complete a short questionnaire including 11 items, each morning after accelerometry measurements (measurement week, see Fig. 2). Participants evaluate the satisfaction with the past night's sleep, current mood and feelings of recreation, difficulties with sleeping in nocturnal awakening and stress level of the past day, on a six-point Likert scale. Information on the presence of nightmares, the consumed quantity of caffeinated beverages and the consumed quantity of alcoholic beverages will be gathered. Additionally, the questionnaire assesses sleeping and wake-up time to double check the ActTrust recordings.

On the last morning of measurements, participants answer the daily questionnaire as well as the Sleep Questionnaire A (SF-A/R)^49^ (measurement week, see Fig. 2). The SF-A/R includes 25 questions assessing several qualitative and quantitative aspects of the past night's sleep. Similarly to the SF-B/R, sleep indices and factor scales can be calculated. We use data from both questionnaires to compare measurements of the self-designed daily sleep questionnaire with a standardised sleep survey.

#### Saliva sampling

To analyse the CAR and the course of alpha-amylase after awakening, saliva samples are collected. We instruct participants to collect saliva samples via Salivette Cortisol (Sarstedt, Nümbrecht, Germany; item number 51.1534.500) devices on three consecutive weekdays (Tuesday to Thursday) at three time points (measurement week, see Fig. 2). The first sampling time is immediately after awakening (base level of CAR), the second sample is collected 30 min after awakening (peak in CAR) and the last sampling time is 45 min after awakening (decrease in CAR). The chosen time intervals conform to the recommended sampling points of the expert consensus guidelines for CAR.^32^

At predetermined times, participants open a container equipped with a medication event monitoring system cap that records the opening time. The Salivettes are equipped with the fitting label for sampling days and time. Participants are instructed in oral and written form not to eat, drink or brush their teeth for 10 mins before collecting saliva samples.

The Salivettes are coded with weekdays and corresponding colours, and are kept separate for each weekday, to maximise clarity and simplify saliva sampling for participants. Sampling times are recorded and participants are informed about the monitoring before the implementation of measurements.

After sample collection, participants store the samples in their fridge or freezer, and return the samples on the second study appointment (T2, see Fig. 2). Then, the saliva samples are stored transitionally in the institute's freezer at −20°C. After completion of the study, the saliva samples are sent with a pseudonymised labelling to the Laboratory of the Department of Biopsychology, Technical University of Dresden, Germany, for analysis.

### Physiological consistency of measurement

As all participants start measurements on a Sunday evening, the weekdays of accelerometry and saliva sampling stay constant over all participants. Measurement timing of female participants is aligned with the female menstrual cycle. Start of the most recent menstruation is assessed at the first study appointment, and all measurements are scheduled for the female participants’ luteal phase, as previous studies found evidence that CAR between women and men might be most similar during the luteal phase.^58,59^ Moreover, use of oral contraceptives is assessed, as this may slightly blunt the CAR.^60^

### Outcomes

#### Primary outcome

The primary outcome of our study is the difference between the index group and control group on all measures of subjective (daily self-assessment questionnaires) and objective (ActTrust measurements, sleep onset latency, sleep efficiency, WASO and awakenings) sleep quality, the pattern of the CAR and the course of alpha-amylase after awakening.

#### Secondary outcome

The secondary outcome of our study is the examination of the severity of depressive symptoms, further psychopathologies, and stress responsiveness at the first study appointment and its relation to sleep quality.

#### *A priori* power calculation

*A priori* sample size was calculated with the program G*Power version 3.1.9.4,^61^ based on a previous study by Schmidt et al^14^ with a medium effect size of *f* = 0.30, an *α* error probability of 0.05 and two groups. To investigate group differences in the CAR through a repeated measures analysis of covariance (ANCOVA), a total of 28 participants would be required to achieve a power of 80%. In accordance with Selvi et al, we assume a medium effect size of *d* = 0.40 for correlational analyses of sleep quality and depressive symptoms.^62^ A sample size of *N* = 44 was deemed necessary for statistical power of 80%. Finally, to enable the calculation of a direction-dependence analysis in case of non-normally distributed and sufficiently skewed data,^63^ we will include a sample of *N* = 60 participants: 30 participants with a depressive disorder and 30 healthy participants as parallel controls.

### Expense allowance

Adolescents participating in our study receive a gift voucher of €50 as an expense allowance for participation.

### Adverse event reporting and harms

We expect no adverse events caused by participating in our study. Medically indicated treatments will not be postponed or changed because of participation. Any serious aggravation of depressive or any other symptoms will result in drop-out and therapeutic assistance if needed. Participants and legal guardians can end study participation at any time without giving reasons, if they no longer wish to participate.

### Statistical analysis

Data will be collected in accordance with the current standard of empirical research. Data will be evaluated with IBM SPSS Statistics version 25.0 for Windows (released 2017; IBM Corp, Armonk, New York). First, depending on the distribution of control variables, differences between the adolescents with depression and the control group will be computed via *t*-tests or Mann–Whitney *U*-tests. As cortisol and alpha-amylase levels are commonly skewed, a square-root transformation will be applied to achieve a normal distribution.^64^ Cortisol and alpha-amylase measurements will be averaged over three days and entered into the repeated measures ANCOVA as the dependent variable, with group as the independent variable.

Cortisol and alpha-amylase increase will be computed by area under the curve analyses.^65^ For an additional measure of increase, delta scores will be computed between baseline and the highest value.^66,67^ To determine group differences in cortisol and alpha-amylase increase, ANCOVAs will be calculated with group as an independent variable.

To examine the relationship between subjective and objective sleep quality, depressive symptoms and cortisol/alpha-amylase increase, bivariate correlations with a false discovery rate correction will be computed.^68^ Effect sizes will be reported with Cohen's *d*, with 0.2 being considered a small, 0.5 a medium and 0.8 a large effect.

Finally, in case of non-normality distributed and sufficiently skewed outcome variables, the direction of the relationship between sleep quality and depressive symptoms will be examined with direction dependence analysis.^63^ Direction dependence analysis enables to statistically test the directional relation between pairs of variables, including observational variables, as well as adjusting for covariates that may contribute to the causal process.^69^

## Discussion

### Knowledge gains

Although sleep disturbance and cortisol release has been the object of broad research in the context of depressive disorders in past years,^70,16^ to the best of our knowledge, no study has examined the combination of subjective sleep quality, accelerometry measuring objective sleep quality, and neurohormonal correlates of HPA axis and autonomous nervous system activity in adolescents with depression before. With the results of our study, we aim to better understand how the strength and course of cortisol and alpha-amylase awakening responses, and subjective as well as objective sleep quality, are related in adolescents with depressive disorders. This present study design allows a wide-ranging approach to sleep impairment and the status of different physiological stress responses. Considering the importance of sleep in the process of growth and maturation, this knowledge is urgently needed to enhance the perspective for adolescent patients with depression, and to integrate sleep quality more profoundly into the treatment process.^34,7,6^

### Relevance of the study

The major value of this study is its contributions toward clinical practice and patients with depressive disorders. In the recruitment process for patients with depression, we receive valuable feedback from both patients and legal guardians about the relevance and level of suffering sleep impairment causes for adolescents with depression. In clinical practice, the urgent demand for targeted assessment strategies and adequate treating to reduce patients’ suffering becomes apparent. Moreover, practising therapists and physicians are frequently confronted with the topic of sleep impairment, but current research on the characteristics of these sleep concerns, the actual amount of objective disruption and subjectively experienced impairment, and especially its effects on different physiological stress systems, does not meet patients’ needs. Previous studies and meta-analyses have repeatedly confirmed these deficits.^6,7,34,71^ The results of the current study are of great value to better our understanding of the origin and overall impact of impaired sleep quality. Moreover, the findings can be a useful reference point for the development of sleep assessments and sleep diaries, which particularly meet the needs of adolescent patients with depressive disorders.

In the pathophysiology regarding trigger and recurrence of depressive disorders, CAR seems to constitute a promising and highly relevant biomarker linked to coping and daily hassles.^22^ An increased CAR in healthy adolescents might be a useful mechanism to adjust to temporarily increased daily demands and to reduce the individual stress level.^22^ A potential dysregulation of this pathomechanism could be highly relevant in the context of stress and occurrence of depressive symptoms.^31^ Because of these previous findings, we added the SSKJ 3-8 R questionnaire to our assessment, which examines vulnerability to daily hassles and the use of various coping mechanisms.^51^

In the context of stress, salivary alpha-amylase has been shown to have a sensitive response to acute stress situations,^22^ with a potentially higher specificity for depressive disorders.^23^

Considering that the onset of depressive symptoms is often linked to a major or minor stressful event,^31^ the examination of pathophysiological aspects of these stress-related biomarkers in adolescents with depressive disorders is essential. By examining the neurohormonal correlates, we aim to contribute to the examination of the relationship between the hormonal system and sleep quality of adolescents with depressive disorders.

All participants in this study will benefit from a detailed and technically supported analysis of sleep quality, which exceeds the diagnostic standard in depressive disorders by far.

### Strengths and limitations

One particular strength of our study is the domestic sleep setting. with its simple and non-invasive accelerometry measurement, which preserves the familiar sleeping habits and allows us to assess sleeping measurements with as little distortion as possible.^9,71^ By choosing out-patients or day patients for the index group, we strive for an undisturbed and natural sleeping condition,^10^ to achieve generalisability of the study findings. Additionally, the combination of an accelerometry measurement and CAR as well as alpha-amylase measurements after awakening provides the benefit of an objective validation of awakening, which is a useful verification for the saliva sampling times.^32,23^ Another strength is the broadness of objective and subjective measurement methods combined with the assessment of both HPA axis and autonomous nervous system activation,^7^ which is, to our knowledge, a rare approach in research focusing on adolescent patients with depression.^24^ With the heterogeneous findings of previous sleep research in adolescent patients with depression^7,11^ and the unexplored potential of alpha-amylase as a useful addition to cortisol-based stress research,^22^ the findings of this study can be of great value to the consideration of depression and physiological stress conditions and its consequences, and may stimulate further research.

A considerable limitation of this study is the exclusion of adolescents with depression who are on antidepressant medication in favour of saliva sampling and including only out-patients and day patients. These eligibility criteria may shift the examined cohort to less severe courses of depressive diseases and exclude long-term and severely ill patients who often take antidepressant medicine or undergo in-patient treatment. Nevertheless, with a prompt study advertisement and a contemporary study appointment, we are able to include a wide range of depression severity before medication intake or in-patient admission is decided. An additional limitation is that external interfering factors disturbing participants’ sleep cannot be controlled in the domestic surrounding during the measurement week. However, these external influences are unavoidable when data is collected in the natural surroundings and under everyday conditions. Subjective sleep impairment in adolescents with depression is mostly experienced within their home environment, thus examining sleep quality, including all external influences, is crucial for a realistic and reliable assessment, and leads to a high ecological validity. Finally, measurements of sleep and salivary biomarkers are limited to 1 week or 3 days, respectively, which implies a certain variability in the course of disease and external influences. Nonetheless, the 3-day saliva sampling and the 1-week sleep measurements constitute a detailed and wide-ranging assessment compared with previous assessments,^30^ and can therefore be considered reliable.

Despite the aforementioned limitations, this study adds crucial and multifactorial value to the pre-existing research in sleep and stress research in adolescents with depressive disorders. In addition, the results of our study could make an important contribution in the establishment of an ambulatory assessment of sleep quality and continuous observation of sleep impairment in the course of disease in adolescents with depressive disorders.

### Trial status

Recruitment has started on 1 February 2021, and the study is intended to be completed by February 2022.

## Data Availability

The data-sets used and/or analysed during the current study are available from the corresponding author, S.K., on reasonable request.

## References

[1] World Health Organization (WHO). *Depression Fact Sheet*. WHO, 2021 (https://www.who.int/en/news-room/fact-sheets/detail/depression).

[2] Cassano P, Fava M. Depression and public health. J Psychosom Res 2002; 53(4): 849–57. 12377293 10.1016/s0022-3999(02)00304-5

[3] Merikangas KR, He J-P, Burstein M, Swanson SA, Avenevoli S, Cui L, Lifetime prevalence of mental disorders in U.S. adolescents: results from the National Comorbidity Survey Replication--Adolescent Supplement (NCS-A). J Am Acad Child Adolesc Psychiatry 2010; 49(10): 980–9. 20855043 10.1016/j.jaac.2010.05.017 PMC2946114

[4] Bertha EA, Balázs J. Subthreshold depression in adolescence: a systematic review. Eur Child Adolesc Psychiatry 2013; 22(10): 589–603. 23579389 10.1007/s00787-013-0411-0

[5] McLeod GFH, Horwood LJ, Fergusson DM. Adolescent depression, adult mental health and psychosocial outcomes at 30 and 35 years. Psychol Med 2016; 46(7): 1401–12. 26818194 10.1017/S0033291715002950

[6] Goldstein TR, Bridge JA, Brent DA. Sleep disturbance preceding completed suicide in adolescents. J Consult Clin Psychol 2008; 76(1): 84–91. 18229986 10.1037/0022-006X.76.1.84 PMC2823295

[7] Rao U. Sleep disturbances in pediatric depression. Asian J Psychiatr 2011; 4(4): 234–47. 22287998 10.1016/j.ajp.2011.09.001 PMC3265574

[8] Lemola S, Ledermann T, Friedman EM. Variability of sleep duration is related to subjective sleep quality and subjective well-being: an actigraphy study. PLoS One 2013; 8(8): e71292. 23967186 10.1371/journal.pone.0071292 PMC3743871

[9] Meltzer LJ, Short M, Booster GD, Gradisar M, Marco CA, Wolfson AR, Pediatric motor activity during sleep as measured by actigraphy. Sleep 2019; 42(1): zsy196. 10.1093/sleep/zsy196 PMC6335874 30335173

[10] McCall WV, Reboussin BA, Cohen W. Subjective measurement of insomnia and quality of life in depressed inpatients. J Sleep Res 2000; 9(1): 43–8. 10733688 10.1046/j.1365-2869.2000.00186.x

[11] Gupta P, Sagar R, Mehta M. Subjective sleep problems and sleep hygiene among adolescents having depression: a case-control study. Asian J Psychiatr 2019; 44: 150–5. 31376799 10.1016/j.ajp.2019.07.034

[12] Fries E, Dettenborn L, Kirschbaum C. The cortisol awakening response (CAR): facts and future directions. Int J Psychophysiol 2009; 72(1): 67–73. 18854200 10.1016/j.ijpsycho.2008.03.014

[13] Adam EK, Kumari M. Assessing salivary cortisol in large-scale, epidemiological research. Psychoneuroendocrinology 2009; 34(10): 1423–36. 19647372 10.1016/j.psyneuen.2009.06.011

[14] Schmidt U, Laessle R, Hellhammer D. Major depression in young girls is related to altered cortisol awakening response. Eur Child Adolesc Psychiatry 2013; 22(6): 379–84. 23292185 10.1007/s00787-012-0371-9

[15] Pruessner JC, Wolf OT, Hellhammer DH, Buske-Kirschbaum A, von Auer K, Jobst S, Free cortisol levels after awakening: a reliable biological marker for the assessment of adrenocortical activity. Life Sciences 1997; 61(26): 2539–49. 9416776 10.1016/s0024-3205(97)01008-4

[16] Stroud CB, Vrshek-Shallhorn S, Norkett EM, Doane LD. The cortisol awakening response (CAR) interacts with acute interpersonal stress to prospectively predict depressive symptoms among early adolescent girls. Psychoneuroendocrinology 2019; 107: 9–18. 31059979 10.1016/j.psyneuen.2019.04.017 PMC6635022

[17] Bhagwagar Z, Hafizi S, Cowen PJ. Increased salivary cortisol after waking in depression. Psychopharmacology (Berl) 2005; 182(1): 54–7. 15991000 10.1007/s00213-005-0062-z

[18] Vreeburg SA, Hoogendijk WJG, van Pelt J, Derijk RH, Verhagen JCM, van Dyck R, Major depressive disorder and hypothalamic-pituitary-adrenal axis activity: results from a large cohort study. Arch Gen Psychiatry 2009; 66(6): 617–26. 19487626 10.1001/archgenpsychiatry.2009.50

[19] Huber TJ, Issa K, Schik G, Wolf OT. The cortisol awakening response is blunted in psychotherapy inpatients suffering from depression. Psychoneuroendocrinology 2006; 31(7): 900–4. 16707227 10.1016/j.psyneuen.2006.03.005

[20] Mangold D, Marino E, Javors M. The cortisol awakening response predicts subclinical depressive symptomatology in Mexican American adults. J Psychiatr Res 2011; 45(7): 902–9. 21300376 10.1016/j.jpsychires.2011.01.001 PMC3270584

[21] Elder GJ, Wetherell MA, Barclay NL, Ellis JG. The cortisol awakening response--applications and implications for sleep medicine. Sleep Med Rev 2014; 18(3): 215–24. 23835138 10.1016/j.smrv.2013.05.001

[22] Nater UM, La Marca R, Florin L, Moses A, Langhans W, Koller MM, Stress-induced changes in human salivary alpha-amylase activity -- associations with adrenergic activity. Psychoneuroendocrinology 2006; 31(1): 49–58. 16002223 10.1016/j.psyneuen.2005.05.010

[23] Bauduin SEEC, van Noorden MS, van der Werff SJA, de Leeuw M, van Hemert AM, van der Wee NJA, Elevated salivary alpha-amylase levels at awakening in patients with depression. Psychoneuroendocrinology 2018; 97: 69–77. 30005283 10.1016/j.psyneuen.2018.07.001

[24] Jezova D, Trebaticka J, Buzgoova K, Durackova Z, Hlavacova N. Lower activity of salivary alpha-amylase in youths with depression. Stress 2020; 23(6): 688–93. 32510266 10.1080/10253890.2020.1777975

[25] Ali N, Pruessner JC. The salivary alpha amylase over cortisol ratio as a marker to assess dysregulations of the stress systems. Physiol Behav 2012; 106(1): 65–72. 22019784 10.1016/j.physbeh.2011.10.003

[26] Fang H, Tu S, Sheng J, Shao A. Depression in sleep disturbance: a review on a bidirectional relationship, mechanisms and treatment. J Cell Mol Med 2019; 23(4): 2324–32. 30734486 10.1111/jcmm.14170 PMC6433686

[27] Kuhlman KR, Chiang JJ, Bower JE, Irwin MR, Seeman TE, McCreath HE, Sleep problems in adolescence are prospectively linked to later depressive symptoms via the cortisol awakening response. Dev Psychopathol 2020; 32(3): 997–1006. 31387652 10.1017/S0954579419000762 PMC7004861

[28] Raniti MB, Allen NB, Schwartz O, Waloszek JM, Byrne ML, Woods MJ, Sleep duration and sleep quality: associations with depressive symptoms across adolescence. Behav Sleep Med 2017; 15(3): 198–215. 26744783 10.1080/15402002.2015.1120198

[29] Buckley TM, Schatzberg AF. On the interactions of the hypothalamic-pituitary-adrenal (HPA) axis and sleep: normal HPA axis activity and circadian rhythm, exemplary sleep disorders. J Clin Endocrinol Metab 2005; 90(5): 3106–14. 15728214 10.1210/jc.2004-1056

[30] van Lenten SA, Doane LD. Examining multiple sleep behaviors and diurnal salivary cortisol and alpha-amylase: within- and between-person associations. Psychoneuroendocrinology 2016; 68: 100–10. 26963376 10.1016/j.psyneuen.2016.02.017 PMC4851910

[31] Dedovic K, Ngiam J. The cortisol awakening response and major depression: examining the evidence. Neuropsychiatr Dis Treat 2015; 11: 1181–9. 25999722 10.2147/NDT.S62289 PMC4437603

[32] Stalder T, Kirschbaum C, Kudielka BM, Adam EK, Pruessner JC, Wüst S Assessment of the cortisol awakening response: expert consensus guidelines. Psychoneuroendocrinology 2016; 63: 414–32. 26563991 10.1016/j.psyneuen.2015.10.010

[33] World Health Organization (WHO). The ICD-10 Classification of Mental and Behavioural Disorders: Clinical Descriptions and Diagnostic Guidelines. WHO, 1992.

[34] Baglioni C, Nanovska S, Regen W, Spiegelhalder K, Feige B, Nissen C, Sleep and mental disorders: a meta-analysis of polysomnographic research. Psychol Bull 2016; 142(9): 969–90. 27416139 10.1037/bul0000053 PMC5110386

[35] Fava M, Alpert JE, Carmin CN, Wisniewski SR, Trivedi MH, Biggs MM, Clinical correlates and symptom patterns of anxious depression among patients with major depressive disorder in STAR*D. Psychol Med 2004; 34(7): 1299–308. 15697056 10.1017/s0033291704002612

[36] Choi KW, Kim Y-K, Jeon HJ. Comorbid anxiety and depression: clinical and conceptual consideration and transdiagnostic treatment. Adv Exp Med Biol 2020; 1191: 219–35. 32002932 10.1007/978-981-32-9705-0_14

[37] Sheehan DV, Lecrubier Y, Sheehan KH, Amorim P, Janavs J, Weiller E, The Mini-International Neuropsychiatric Interview (M.I.N.I.): the development and validation of a structured diagnostic psychiatric interview for DSM-IV and ICD-10. J Clin Psychiatry 1998; 59(suppl 20): 22–33, quiz 34–57. 9881538

[38] Hautzinger M, Keller F, Kühner C. *Beck-Depressions-Inventar Revision [BDI-II], 2nd Auflage* *[Beck Depression-Inventory Revision [BDI-II]].* Pearson Assessment, 2009.

[39] Richter P, Werner J, Heerlein A, Kraus A, Sauer H. On the validity of the Beck Depression Inventory. A review. Psychopathology 1998; 31(3): 160–8. 9636945 10.1159/000066239

[40] Kühner C, Bürger C, Keller F, Hautzinger M. Reliabilität und Validität des revidierten Beck-Depressions-Inventars (BDI-II) Befunde aus deutschsprachigen Stichproben. [Reliability and validity of the Revised Beck Depression Inventory (BDI-II). Results from German samples] Nervenarzt 2007; 78(6): 651–6. 16832698 10.1007/s00115-006-2098-7

[41] Dolle K, Schulte-Körne G, O'Leary AM, von Hofacker N, Izat Y, Allgaier A-K. The Beck Depression Inventory-II in adolescent mental health patients: cut-off scores for detecting depression and rating severity. Psychiatry Res 2012; 200(2–3): 843–8. 22657953 10.1016/j.psychres.2012.05.011

[42] Pietsch K, Hoyler A, Frühe B, Kruse J, Schulte-Körne G, Allgaier A-K. Früherkennung von Depressionen in der Pädiatrie: Kriteriumsvalidität des Beck Depressions-Inventar Revision (BDI-II) und des Beck Depressions-Inventar-Fast Screen for Medical Patients (BDI-FS). [Early Detection of Major Depression in Paediatric Care: Validity of the Beck Depression Inventory–Second Edition (BDI-II) and the Beck Depression Inventory–Fast Screen for Medical Patients (BDI-FS)]. Psychother Psychosom Med Psychol 2012; 62(11): 418–24. 22723284 10.1055/s-0032-1314869

[43] Margraf J, Ehlers A. *Beck-Angst-Inventar [BAI] 1st Auflage* *[Beck Anxiety Inventory [BAI]].* Pearson Assessment, 2007.

[44] Beck AT, Epstein N, Brown G, Steer RA. An inventory for measuring clinical anxiety: psychometric properties. J Consult Clin Psychol 1988; 56(6): 893–7. 3204199 10.1037//0022-006x.56.6.893

[45] Geissner E, Huetteroth A. Beck Anxiety Inventory Deutsch – ein reliables, valides und praxisgeeignetes Instrument zur Messung klinischer Angst [Beck Anxiety Inventory German Version - A Reliable, Valid, Patientfriendly Instrument for Measuring Clinical Anxiety]. Psychother Psychosom Med Psychol 2018; 68(3–4): 118–25. 29351711 10.1055/s-0043-122941

[46] Osman A, Hoffman J, Barrios FX, Kopper BA, Breitenstein JL, Hahn SK. Factor structure, reliability, and validity of the Beck Anxiety Inventory in adolescent psychiatric inpatients. J Clin Psychol 2002; 58(4): 443–56. 11920696 10.1002/jclp.1154

[47] Hamann C, Rusterholz T, Studer M, Kaess M, Tarokh L. Association between depressive symptoms and sleep neurophysiology in early adolescence. J Child Psychol Psychiatry 2019; 60(12): 1334–42. 31512761 10.1111/jcpp.13088

[48] Kandel DB, Davies M. Epidemiology of depressive mood in adolescents: an empirical study. Arch Gen Psychiatry 1982; 39(10): 1205–12. 7125850 10.1001/archpsyc.1982.04290100065011

[49] Görtelmeyer R. *Schlaffragebogen A und B: SF-A/R und SF-B/R, 1st Auflage* *[Sleep Questionnaires A and B: SF-A/R and SF-B/R].* Hogrefe, 2011.

[50] Lohaus A, Eschenbeck H, Kohlmann C-W, Klein-Heßling J. *Fragebogen zur Erhebung von Stress und Stressbewältigung im Kindes- und Jugendalter (SSKJ 3-8)* *[Questionnaire for the Measurement Stress and Coping in Children and Adolescents (SSKJ 3-8)].* Hogrefe, 2006.

[51] Weis M, Heine J-H. Assessing emotion regulation strategies in Chile: a Spanish language adaptation of the German SSKJ 3-8 scales. Front Psychol 2019; 10: 2870. 31998177 10.3389/fpsyg.2019.02870 PMC6962141

[52] Schmidt U, Laessle R. Stressbezogene Determinanten für die Aufrechterhaltung von Depressionen bei Mädchen [Stress-related risk factors for depression in girls]. Z Kinder Jugendpsychiatr Psychother 2014; 42(3): 157–66. 24846864 10.1024/1422-4917/a000285

[53] Eschenbeck H, Kohlmann C-W, Lohaus A, Klein-Heßling J. Die Diagnostik von Stressbewältigung mit dem “Fragebogen zur Erhebung von Stress und Stressbewältigung im Kindes- und Jugendalter” (SSKJ 3-8) [The assessment of coping with the “Questionnaire for the Measurement Stress and Coping in Children and Adolescents (SSKJ 3-8)”: Factord and psychometric analyses]. Diagnostica 2006; 52(3): 131–42.

[54] Watzlawik M. Die Erfassung des Pubertätsstatus anhand der Pubertal Development Scale [Assessing pubertal status with the Pubertal Development Scale: First steps towards an evaluation of a German translation]. Diagnostica 2009; 55(1): 55–65.

[55] Petersen AC, Crockett L, Richards M, Boxer A. A self-report measure of pubertal status: reliability, validity, and initial norms. J Youth Adolesc 1988; 17(2): 117–33. 24277579 10.1007/BF01537962

[56] Ruiz FS, Beijamini F, Beale AD, Gonçalves B, Vartanian D, Taporoski TP, Early chronotype with advanced activity rhythms and dim light melatonin onset in a rural population. J Pineal Res 2020; 69(3): e12675. 32598502 10.1111/jpi.12675 PMC7508839

[57] Rodrigues J, Eckeli AL. Validação de um actígrafo nacional [Validation of a national actigraph]. *D Medical doctoral thesis* RNP Neurologia, Psiquiatria e Psicologia Medica, Universidade de São Paulo, 2018.

[58] LeRoux A, Wright L, Perrot T, Rusak B. Impact of menstrual cycle phase on endocrine effects of partial sleep restriction in healthy women. Psychoneuroendocrinology 2014; 49: 34–46. 25051527 10.1016/j.psyneuen.2014.06.002

[59] Benz A, Meier M, Mankin M, Unternaehrer E, Pruessner JC. The duration of the cortisol awakening pulse exceeds sixty minutes in a meaningful pattern. Psychoneuroendocrinology 2019; 105: 187–94. 30595408 10.1016/j.psyneuen.2018.12.225

[60] Bouma EMC, Riese H, Ormel J, Verhulst FC, Oldehinkel AJ. Adolescents’ cortisol responses to awakening and social stress; effects of gender, menstrual phase and oral contraceptives. The TRAILS study. Psychoneuroendocrinology 2009; 34(6): 884–93. 19195792 10.1016/j.psyneuen.2009.01.003

[61] Faul F, Erdfelder E, Buchner A, Lang A-G. Statistical power analyses using G*Power 3.1: Tests for correlation and regression analyses. Behav Res Methods 2009; 41: 1149–60. 19897823 10.3758/BRM.41.4.1149

[62] Selvi Y, Aydin A, Boysan M, Atli A, Agargun MY, Besiroglu L. Associations between chronotype, sleep quality, suicidality, and depressive symptoms in patients with major depression and healthy controls. Chronobiol Int 2010; 27(9–10): 1813–28. 20969525 10.3109/07420528.2010.516380

[63] Wiedermann W, von Eye A. Direction-dependence analysis. Int J Behav Dev 2015; 39(6): 570–80.

[64] Kobayashi H, Miyazaki Y. Distribution characteristics of salivary cortisol measurements in a healthy young male population. J Physiol Anthropol 2015; 34(1): 30. 10.1186/s40101-015-0068-0 PMC4543482 26286592

[65] Pruessner JC, Kirschbaum C, Meinlschmid G, Hellhammer DH. Two formulas for computation of the area under the curve represent measures of total hormone concentration versus time-dependent change. Psychoneuroendocrinology 2003; 28(7): 916–31. 12892658 10.1016/s0306-4530(02)00108-7

[66] Kudielka BM, Buske-Kirschbaum A, Hellhammer DH, Kirschbaum C. HPA axis responses to laboratory psychosocial stress in healthy elderly adults, younger adults, and children: impact of age and gender. Psychoneuroendocrinology 2004; 29(1): 83–98. 14575731 10.1016/s0306-4530(02)00146-4

[67] Kudielka BM, Schommer NC, Hellhammer DH, Kirschbaum C. Acute HPA axis responses, heart rate, and mood changes to psychosocial stress (TSST) in humans at different times of day. Psychoneuroendocrinology 2004; 29(8): 983–92. 15219648 10.1016/j.psyneuen.2003.08.009

[68] Hochberg Y, Benjamini Y. More powerful procedures for multiple significance testing. Stat Med 1990; 9(7): 811–8. 2218183 10.1002/sim.4780090710

[69] Wiedermann W, Li X. Direction dependence analysis: a framework to test the direction of effects in linear models with an implementation in SPSS. Behav Res Methods 2018; 50(4): 1581–601. 29663299 10.3758/s13428-018-1031-x

[70] Lopez-Duran NL, Kovacs M, George CJ. Hypothalamic-pituitary-adrenal axis dysregulation in depressed children and adolescents: a meta-analysis. Psychoneuroendocrinology 2009; 34(9): 1272–83. 19406581 10.1016/j.psyneuen.2009.03.016 PMC2796553

[71] Lofthouse N, Gilchrist R, Splaingard M. Mood-related sleep problems in children and adolescents. Child Adolesc Psychiatr Clin N Am 2009; 18(4): 893–916. 19836695 10.1016/j.chc.2009.04.007

